# Usefulness of skin tests in penicillin-allergic patients after cephalosporins challenge

**DOI:** 10.1186/2045-7022-4-S3-P64

**Published:** 2014-07-18

**Authors:** Sergio Testi, Giuseppe Ermini, Maria Loredana Iorno, Donatella Macchia, Stefania Capretti, Maurizio Giuseppe Severino

**Affiliations:** 1 Azienda Sanitaria di Firenze, San Giovanni di Dio Hospital, Allergy and Clinical Immunology Unit, Italy

## Background

Subjects with a history of documented penicillin allergy but with negative skin test results for cephalosporins, can tolerate subsequent challenge doses of cephalosporin without an allergic reaction. [1]. In immediate reactions to betalactams (BL), in some instances, specificity was mainly directed to the BL inducing the reaction, but in other cases specificity was mainly directed to another structure, probably related with previous BL exposure [2].

## Methods

Between November 2011 and October 2013, we prospectively recruited a total population of 100 (71 women, 29 men) patients from the outpatients of the Clinical Immunology Unit (Florence, Italy). Patients had a history of immediate reactions to at least one penicillin (Tab. 1) and positive results on skin tests for one or more penicillin reagents (penicilloyl-polylysine, minor determinant mixture, and benzylpenicillin), one or more semi-synthetic penicillins (amoxicillin, ampicillin), or both [3]. We also evaluated sensitization to cephalosporins by using skin tests [4] with second-generation (cefuroxime), and third-generation (ceftazidime, ceftriaxone, and cefotaxime) cephalosporins. In cases of negative results for all of these cefalosporins, cefuroxime and ceftriaxone were administered to consenting patients [1].

## Results

74 of the 100 patients underwent challenges with oral cefuroxime axetil and intramuscular ceftriaxone: all tolerated the challenge. After about a month we repeated the skin tests with PPL, MDM, and the two cephalosporins administered (cefuroxime and ceftriaxone). 8 patients (10.8%) presented new sensitizations: 2 for MDM, 2 for PPL and MDM, 1 for cefuroxime, 1 for ceftriaxone, 1 for PPL and ceftriaxone, 1 for PPL, MDM, cefuroxime and ceftriaxone. 26 patients refused the double challenge and only underwent a tolerance to cefuroxime axetil test. In one of these the challenge was positive. The skin tests after a month with PPL, MDM, and the cephalosporin administered (cefuroxime) presented new sensitizations in only one patient (4.0%), for PPL.

## Conclusion

One month after the challenge, the percentage of sensitization seems to be higher in patients who underwent tolerance tests with two cephalosporins. It would be useful to have more data available to confirm the above mentioned assumptions, but above all it would be useful to know if we can recommend the cephalosporins tolerated with negative skin tests after a month, even if associated with positive PPL and/or MDM and/or other cephalosporin administered with the tolerance test.

